# Exploring Trust Formation and Antecedents in Social Commerce

**DOI:** 10.3389/fpsyg.2021.789863

**Published:** 2022-01-28

**Authors:** Ali Alkhalifah

**Affiliations:** Department of Information Technology, College of Computer, Qassim University, Buraydah, Saudi Arabia

**Keywords:** social commerce (SC), electronic commerce, trust, social media, online payment, online community

## Abstract

With the rapid increase in social media users and netizens globally, the proclivity for online shopping using social commerce (SC) platforms cannot be ignored. Trust has been recognised as a constant challenge in the context of social commerce due to the lack of face-to-face interaction. Therefore, there is a dire need to enhance the trust of consumers in social commerce platforms. However, the research in the formation of trust in social commerce and antecedents remains limited. In addition, the existing SC research failed to include its multidimensional view to investigate user behaviour. This study fills this gap and extends existing knowledge by developing a model exploring the antecedents of trust in social commerce. Drawing upon the social-technical theory and trust lens, this study attempts to identify the role of (i) structural assurance (SA) and SC platforms as an institution-based trust, (ii) trust in sellers and trust in SC community as trusting beliefs, and (iii) trust in online payment as a cognitive trust on trust and intention of the social commerce. This research employs a dataset (*n* = 406) collected using an online survey; the research subjects were recruited from Australia, the United States, and the United Kingdom. This study uses the partial least squares structural equation modeling (PLS-SEM) approach to analyse the data and to confirm the hypothesis proposed in the research model. The empirical findings show that trust in social commerce influences behavioural intention. In addition, trust in the SC platform, the SC community, and online payment influence the trust in SC. Likewise, SA and trust in the SC platform have a significant relationship with trust in sellers, the SC community, and online payment. Finally, this study discusses the theoretical contributions and practical insights to several limitations and suggests directions for future research.

## Introduction

Social media sites such as Instagram, Twitter, and Facebook have become ubiquitous, creating possibilities for electronic business models usually known as social commerce (SC). Social commerce provides new opportunities for incorporating social inputs into financial operations (Mamonov and Benbunan-Fich, 2017). Social commerce is an innovative approach toward doing business by employing social media for networking (Sohn and Kim, 2020; Yang et al., 2021). Although social commerce is novel in the field of e-commerce, it is rapidly advancing and drawing the interest of practitioners and researchers (Lin et al., 2017; Hajli, 2020; Tuncer, 2021). Social commerce has become a substantial area of study that has received much attention in recent years (Lin et al., 2017; Sarkar et al., 2020; Molinillo et al., 2021; Mou and Benyoucef, 2021; Yang, 2021).

Progressing to social commerce from e-commerce has helped in the role reversal of sellers and buyers, where the bargaining power is now in the hands of the latter (Huang and Benyoucef, 2013; Hajli and Sims, 2015; Wongkitrungrueng and Assarut, 2020). Social commerce has transformed e-commerce from a product to a customer-based platform (Huang and Benyoucef, 2013; Lin et al., 2019; Leong et al., 2020). Moreover, social commerce is remarkably flourishing. For instance, the Chinese social commerce industry will hit $351.65 billion by the end of 2021 (Enberg, 2021), while SC in the United States is expected to grow by 34.8% to $36.09 billion in 2021 (Lipsman, 2021). Furthermore, the percentage of social commerce consumers in the United States increased by 25.2% to 80.1 million in 2020, and by 2021, it is expected to increase by another 12.9% to 90.4 million (Lipsman, 2021). Similarly, according to research conducted by Gartner, 66% of the 424 businesses under study employed social commerce practices (O’Shea, 2018).

There might be a shift of trust to social commerce from e-commerce (Chen and Wang, 2016; Hajli, 2020). Trust, which has been identified as one of the most significant barriers to e-commerce, is an even greater issue in social commerce (Lin et al., 2017, 2019; Sarkar et al., 2020; Tuncer, 2021). Where e-commerce platforms reinforce platform-based trust and security, social media platforms do not take responsibility for the social commerce posts and comments of buyers (Mou and Benyoucef, 2021; Tuncer, 2021). Hence, boosting the trust of consumers in the SC platforms is the need of the hour. Customer reviews, likes, comments, and communication with the seller in social commerce build the trust of potential customers (Sohn and Kim, 2020). For instance, Instagram is a social media platform based on pictures that facilitates the engagement between sellers and buyers and is conducive to genuine purchasing (Molinillo et al., 2021). Since trust is a significant component in customer purchase intentions in social commerce, practitioners will benefit from a deeper grasp of the subject.

Moreover, social commerce decision making that is related to purchases and transactions is greatly affected by researching trust models and influences that are becoming imperative (Hajli, 2020; Lãzãroiu et al., 2020; Tuncer, 2021). However, the factors that influence trust in social commerce are primarily unknown (Lin et al., 2017; Leong et al., 2020, 2021; Sarkar et al., 2020; Tuncer, 2021). Previous research has viewed trust in social commerce from a single dimension, emphasising trust in sites (Lin et al., 2019). Little attention has been devoted to trust regarding the seller and the SC platforms (Wongkitrungrueng and Assarut, 2020; Mou and Benyoucef, 2021; Tuncer, 2021). In addition, very few have investigated the trust of customers in sellers on SC platforms influencing their purchasing behaviour (Tuncer, 2021). Furthermore, few studies have looked at the institutional-based trust, for instance, structural assurance (SA) (Sarkar et al., 2020), trust in social commerce applications, and websites (Luo et al., 2010; Hajli, 2020).

Trust is an important factor in the purchasing behaviour of customers. Thus, trust is a multifaceted concept in e-commerce that includes trust in the supplier, website, and community members (Abed, 2018; Fu et al., 2018). Trusting social commerce is viewed as a one-dimensional or multidimensional notion (Lin et al., 2019; Mou and Benyoucef, 2021; Tuncer, 2021). Social commerce encompasses people, information, technology, and management (Wang and Zhang, 2012). Likewise, additional research shows that social commerce incorporates various technological, social, and commercial elements (Wang and Zhang, 2012; Huang and Benyoucef, 2013). According to this viewpoint, the nature of a multifaceted trust also relates to customer trust in social commerce (Lin et al., 2019). Nevertheless, previous research has ignored this notion, and a few social commerce studies have investigated trust as a multifaceted construct (Lin et al., 2019; Mou and Benyoucef, 2021).

According to our in-depth research, the study done by Lin et al. (2019) was the foremost and only previous research assessing social commerce trust from a multifaceted viewpoint that is encompassing trust in e-commerce websites, social media, SC features, and consumers. The study defined trust in SC as a second factor construct and assessed its impact on the purchase behaviour of an e-commerce site. Their study, however, did not identify nor evaluate the connections between these trust variables. Therefore, this study addresses these gaps by introducing a new conceptualisation of trust in SC and investigating the origins of trust in social commerce, such as institutional-based trust (SA and SC platforms), trusting beliefs (trust in sellers and community members), and cognitive trust (online payment), along with the relationship between these trust variables. As a multidimensional factor, this study analyses both trust in sellers and the SC platform. This research looks at the following question:

RQ. In social commerce, which dimension of trust is more linked with customer behaviour?

On the basis of social-technical theory (Bostrom and Heinen, 1977), this study identifies social commerce trust from a multifaceted perspective (the technology and people facets), aligned with the trust theory from previous research (McKnight et al., 2002; Kim et al., 2005; Lin et al., 2019).

This paper is structured in the following way. First, an outline of social commerce, the social-technical theory, and trust is provided. Second, social commerce trust formation is presented. The research model and hypothesis formulation are discussed in the fourth section. Following that, the research technique used in this study and the data analysis outcomes are explained. Finally, we discuss the implications and limitations of the study, along with future research prospects.

## Literature Review

### Social Commerce

Since the social commerce concept is broad and is associated with several inconsistencies, including activities of e-commerce that involve social networks and social media, it currently lacks a standard definition (Sohn and Kim, 2020). According to Stephen and Toubia (2010), social commerce is a type of Internet-based social network that enables people to directly engage in the selling and marketing of products or services *via* websites and online communities. Social commerce is a term used to define an e-commerce practice involving social media and social networks (Liang et al., 2011; Mamonov and Benbunan-Fich, 2017). In addition, social commerce entails using Web 2.0 technologies to promote online social engagements and customer experience to help buyers purchase goods and services (Kim and Park, 2013; Yang et al., 2021). Sohn and Kim (2020) also define social commerce as “selling goods through SNSes as well as the electronic commerce based on a specific site.” We use the following definition of social commerce by Meilatinova (2021) in this study, “as an extension of e-commerce sites, integrated with social media and Web 2.0 technology to encourage online purchases and interactions with customers before, during, and after the purchase.”

The literature has identified two kinds of social commerce. The first is social networking websites incorporating tools to facilitate online transactions. The second is typical e-commerce websites incorporating elements such as like, share, and comment to encourage social networking (Liang et al., 2011; Zhang and Benyoucef, 2016; Hajli et al., 2017; Mou and Benyoucef, 2021). However, other social commerce categories can be established based on the combination of characteristics (Molinillo et al., 2021). Based on how consumer interactions are built, what their aims are, what information they exchange, and the sort of platform they utilise, Han et al. (2018), for example, identified eight distinct forms of social commerce.

A lot of attention has been given to social commerce (Lin et al., 2017; Mou and Benyoucef, 2021). Issues such as engagement in SC websites and sellers (Wongkitrungrueng and Assarut, 2020), information sharing (Hajli et al., 2017), value co-creation (Tajvidi et al., 2021), decision making in SC regarding purchase (Chen et al., 2017; Lãzãroiu et al., 2020), and the role of peers relations and social influence (Yang, 2021) are investigated in prior research. Electronic word of mouth (eWOM) (Cheng et al., 2021) and features of social commerce Web design (Molinillo et al., 2021) are also discussed in previous studies. However, trust and its formation have been paid little attention to in previous SC research (Leong et al., 2020, 2021; Meilatinova, 2021; Mou and Benyoucef, 2021).

### Social-Technical Theory

**FIGURE 1:**
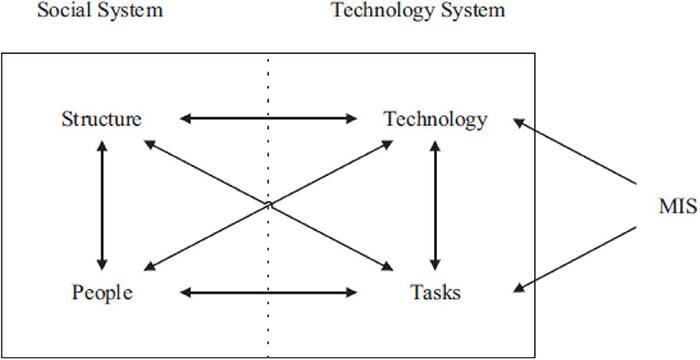
Social-technical theory (Bostrom and Heinen, 1977).

According to social-technical theory (Figure 1), a system may be divided into social and technical subsystems (Bostrom and Heinen, 1977). In addition to this, the procedures and technologies make the technical subsystem, and these systems allow users to perform multiple functions. For example, users can convert inputs into outputs and do some predefined system activities. On the other hand, the social subsystem consists of knowledge, users, connections, values, and a mechanism of reward (Lin et al., 2019). The subsystems mentioned above are not isolated; instead, they are related and should function in tandem for optimal performance of the system (Bostrom and Heinen, 1977; Bostrom et al., 2009). According to this viewpoint, people and technology play essential parts in determining the functionality of the system since they mainly define the connection between the two subsystems (Kong et al., 2020).

This theory is relevant to social commerce, directed by web technology and the people involving the aforementioned subsystems (Kong et al., 2020). The social commerce subsystem consists of people (social media users, sellers, and buyers) and organisational components (groups, communities, and fan pages) supporting the social relationships and interactions, facilitating social support, and making people rely on and commit to an organisation (Bostrom et al., 2009; Liang et al., 2011; Wang and Zhang, 2012; Ng, 2013; Lin et al., 2019). The technological subsystem of social commerce comprises Web technology, including social media, social commerce elements, and e-commerce sites and process elements that support the SC operations like commercial information exchange and consumer evaluations (Liang et al., 2011; Wang and Zhang, 2012).

In addition, technological variations do not restrict the technical system and do not influence the system and the users of the social structure, as stated by the social-technical theory (Lin et al., 2019). E-commerce sites alter the technological environment by including SC features and social networking sites. As a result, the affordances that allow customers to do new activities are provided (i.e., new customer behaviours like information sharing about the product). Moreover, such modifications can also influence people and the structure of the task (i.e., examining how customers engage on online e-commerce sites and online platforms) (Bostrom et al., 2009). This subsystem also forms the basis for the social subsystem in social commerce. Finally, the internet and online sites also allow individuals to connect, build, and sustain social ties.

In contrast, progression in the technical subsystem of social commerce may be triggered by the social subsystem because organisations and people require valuable support, interactions, and social relationships. This can be obtained through technological capabilities (Kong et al., 2020). These two subsystems have been demonstrated to operate well together in social commerce (Lin et al., 2019). As a result, these two subsystems benefit customers by enhancing their shopping experience and purchasing decisions (Lin et al., 2019). Hence, the social-technical theory is an ideal theoretical framework since it may aid in understanding how many variables interact to build the trust of customers in social commerce. Using social–technical theory, it has been suggested that technology and people are the fundamental elements that propel social commerce and can truly depict the social commerce subsystems (Lin et al., 2019; Kong et al., 2020).

This study examines a social commerce platform setting that combines social networking sites with conventional e-commerce websites. Moreover, this study encourages the SC attributes, such as consumer evaluations and sharing on social commerce sites. Our primary focus is on community members and sellers who have provided the above-mentioned SC features. Hence, the people component is represented through the SC community members and sellers because they are the key competence of these SC features. Social commerce platforms, SA, and online payment best represent the technology. The technology and people dimensions are further discussed in sections “Institutional-Based Trust” and “Trusting Beliefs.”

### Trust

Trust has been thoroughly researched and distinctly defined in several research papers. Due to the diversity of definitions for this perplexing concept, we opted to utilise a well-referenced and widely accepted definition of trust that has been cited in several pieces of research. The definition of trust by Mayer et al. (1995) has been used in this study, i.e., “a party’s willingness to be responsible for the acts of another party in exchange for the anticipation that others will perform a specific action significant to the truster, regardless of the truster’s ability to track or control the other party” (p. 712).

Trust is important in trade interactions, particularly those with unknown dangers in the Internet world (Gefen et al., 2003). The acceptance or rejection of consumers of online transactions is heavily influenced by their trust (Pavlou and Gefen, 2004). Trust is frequently interpreted as confidence in both user feedback and the capability, reliability, and honesty of retail suppliers (Hajli et al., 2017; Leong et al., 2020). Trust is a three-dimensional concept encompassing competence, integrity, and benevolence (McKnight et al., 2002). Competence means the capability of the trustee to meet the requirements of the truster; benevolence is the concern of the trustee’s for and determination to behave in the interests of the truster; integrity is defined as the capability of the trustee to keep honest commitments (McKnight et al., 2002). The trustor is the person who creates the trust, while the trustee is the one who receives it (McKnight et al., 1998).

This study responded to the appeal from previous studies (Lin et al., 2017, 2019; Hajli, 2020; Leong et al., 2020, 2021; Mou and Benyoucef, 2021; Tuncer, 2021) for more research on the factors contributing to trust in the SC context. They proposed that trust is the notion that enables customers to adopt SC after considering the features of social media and vendor suppliers, the underlying Internet infrastructure, and the payment.

### Trust in Social Commerce

Previous SC studies have recognised that trust is a significant factor that drives the behavioural intentions of customers like intention to use (Rahman et al., 2020), buying or repurchasing intent (Kim and Park, 2013; Hajli et al., 2017; Meilatinova, 2021), and aim to support (Tuncer, 2021), word of mouth (WOM) (Meilatinova, 2021), and loyalty behaviour intention (Molinillo et al., 2021). There is limited studies that emphasise SC trust and behaviour usage intention (Mou and Benyoucef, 2021; Tuncer, 2021).

Additionally, a lack of empirical investigations demonstrates the trust antecedents in SC (Lin et al., 2017, 2019; Leong et al., 2020, 2021; Meilatinova, 2021). The social factors of trust have been extensively researched (Kim and Peterson, 2017; Leong et al., 2020; Mou and Benyoucef, 2021). For instance, Leong et al. (2020) investigated trust in social commerce using networks and social presence. Their study discovered that information support had the most significant influence on the SC trust using a mixed SEM-ANN method. According to Leong et al. (2020), “the drivers of trust creation in e-commerce remain substantially unknown.” Recently, Mou and Benyoucef (2021) published meta-analysis research on customer behaviour in SC. It analyses several theoretical frameworks and examines the influence on the phases of consumer decision-making of the elements provided by them and the moderators among the variables. Interpersonal and organisational trust was found to be associated with SC behaviour. Additionally, a meta-analysis examines the factors contributing to mobile consumer engagement trust (Sarkar et al., 2020). The study found several influential elements that affect trust in mobile commerce. These elements include, but are not limited to, SA, disposition to trust, perceived security and risk, information and user interface quality, perceived usefulness, and ease of use.

In the SC literature, many trust factors have been identified. As an example, these variables include trust in SC websites and applications (Hajli, 2020), trust in sellers and social media platforms (Hajli et al., 2017; Tuncer, 2021), community trust (Cheng et al., 2019; Molinillo et al., 2021), perceived trust (Kim and Park, 2013; Leong et al., 2020), trust in mobile social commerce (Leong et al., 2021), and trust disposition (Cheng et al., 2019).

Trust was found as a one-dimensional component in most of these investigations. There is a lack of research that has seen several distinct trust variables in a single study (Lin et al., 2019; Hajli, 2020; Mou and Benyoucef, 2021; Tuncer, 2021). Additionally, to our knowledge, only one study conceptualises trust as a multidimensional component, namely, Lin et al. (2019). Their study employed the social-technical theory to conceptualise the SC trust in a multidimensional view. Social commerce trust construct included trust in e-commerce sites, trust in social media, SC features, and consumers. The analysis of data of 904 United States Amazon customers, collected by using an online survey, shows a strong provision for this conceptualisation. In addition, the results revealed that trust in social commerce features and trust in consumers have stronger impacts than trust in social media and trust in e-commerce sites in the formation of SC trust. Moreover, Lin et al. (2019) believe that “understanding the role and antecedents of customer trust in social e-commerce would give additional insight into the phenomena of social commerce and its economic effects.” Therefore, this study addresses these gaps by identifying diverse trust variables, including trust in the Internet (SA), trust in SC platforms (social networking and e-commerce sites) (multidimensional), trust in sellers (multidimensional), trust in SC community members, and cognitive trust (online payment). The next section discusses the trust constructions used in this study.

### Social Commerce Trust in This Study

There is a significant degree of ambiguity from an SC view because of the lack of face-to-face interactions and the massive amount of user-generated material (Featherman and Hajli, 2016). As a result, building relationships with SC users is challenging as they cannot verify the reliability and authenticity of the user-generated material (Leong et al., 2020). Additionally, because the SC customers lack face-to-face contact, they cannot detect non-verbal facial movements, making trust-building in SC problematic. As a result, it is critical to understand trust in social commerce better.

Trust has long been seen as a critical component of the activity of customers in e-commerce. Trust in e-commerce is a multifaceted concept comprised of three parts: (1) trust in website, (2) trust in network operator, and (3) trust in community members (Abed, 2018; Fu et al., 2018). However, in SC notion, utilising a part of the Chinese population, it was discovered that trust in community members had no significant effect on social purchasing intent. In comparison, a poll of Netizens of the Douban.com portal discovered that trust in community members has a significant role in influencing social shopping intent (Mou and Benyoucef, 2021). This motivated us to investigate if one component of trust is more important than the others in influencing customer behaviour in social commerce, or if the effects of trust varied across different SC platforms (Mou and Benyoucef, 2021).

**FIGURE 2:**
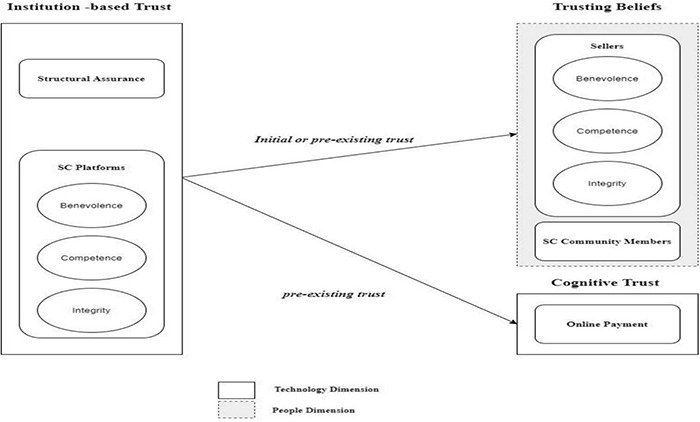
Social commerce trust formation.

Figure 2 depicts the SC trust model established in this study. This model has three concept levels: (1) trusting beliefs, (2) institutional-based trust, and (3) cognitive trust. It is essential to distinguish between trust in an online service provider and faith in technology as a transaction platform (McKnight et al., 2002; Luo et al., 2010). Therefore, the model distinguishes between two sorts of trust: initial trust in the sellers, SC community members (which may be new or pre-existing trust), pre-existing trust in the SA, SC platforms, and online payment. Trust is assessed between the two distinct exchange transaction parties, as the transaction duties of the social commerce platform and sellers may vary per customer. As a result, the trust of each consumer in the SC platform and other customers may vary.

Trust in social commerce or SC trust is defined as the user’s beliefs that SC systems are reliable, and a feeling of being protected and being concerned in the online purchasing transaction (Ng, 2013; Leong et al., 2020). The phrase “trusting beliefs” refers to the opinion of a confident truster that the trustee – such as a particular Web-based vendor – possesses favourable characteristics for the truster (McKnight et al., 2002). Both trusts in sellers and in SC community members are considered to be the steadfast beliefs in this study. Trust in the sellers refers to the expectation of consumers to be able to trust the official testimony and speech of the sellers on the SC platforms (Tuncer, 2021). The term “SC community” refers to a subset of social media and online members who utilise user-generated content (UGC) and human engagement to assist customers in making product and service purchase decisions (Cheng et al., 2021). Opinions of consumers of the integrity and capability of the SC platforms to provide accurate evaluations and suggestions are described as trust in digital spaces (McKnight, 2005).

As per the model of trust-based acceptance by Komiak and Benbasat (2006), cognitive trust captures the unique trusting views of users about the trustworthy qualities of the trustee, such as professional qualifications, familiarity, and dependability. Intellectual trust will be created to a high degree if people find adequate causes to trust (Gong et al., 2020). Cognitive trust in online purchases is used in this study to refer to the belief of users in the potential of SC platforms to offer accurate and trustworthy financial services (Gong et al., 2020; Leong et al., 2021). It measures the cognitive views of consumers about the dependability and credibility of online payment systems. Trust has been recognised as a critical element in the research of online human behaviour, particularly in transactions like SC, where customers are more receptive to online payments (Williams, 2021).

Trust in the Internet and SC platforms is the pre-existing trust based on institutions (Hajli, 2020; Tuncer, 2021). Institutional trust is defined as “the assumption that necessary structural circumstances exist (e.g., on the Internet) to increase the chance of success in an endeavour such as e-commerce” (McKnight et al., 2002, p. 339). Trust in institutions (through the Internet) is described as having a single dimension: SA. SA is “belief in the existence of structures like as guarantees, rules, pledges, legal remedies, or other mechanisms designed to encourage success” (McKnight et al., 2002, p. 339). A customer with a high level of structural certainty regarding the Internet would think that technical and legal safeguards, such as data security, would protect him/her from financial, privacy, or identity information loss. The SC users feel that SC sites on the Internet are competent and trustworthy. The term “trust in the social commerce platform” relates to the views of consumers of the institutional structure of social media and e-commerce platforms system, and their sentiments about the SA of the system (Pavlou and Gefen, 2004; Tuncer, 2021).

#### The People Dimensions

The critical components of people supporting social interactions and relationships in social commerce are the sellers, online community members, and features (Wang and Zhang, 2012; Lin et al., 2019; Wongkitrungrueng and Assarut, 2020). According to Hajli (2020), customer trust in the seller is becoming ever more important as a prerequisite for consumer shopping, assessments, and purchase decisions. Trust may be formed through the security of the atmosphere established by the place of business or the e-commerce site of the seller. Furthermore, in the social commerce environment, to this viewpoint, sellers and online communities are the key contributors to social commerce (Hajli, 2020; Cheng et al., 2021). A highly interactive platform is provided to the customers by sellers and online communities in social commerce. They can engage with one another and share their opinions on a service or product (Liang et al., 2011; Leong et al., 2020). During the purchase procedure, however, their relationship with the SC consumers differs. Sellers and online communities are also important in social commerce literature (e.g., Ng, 2013; Cheng et al., 2019, 2021; Lin et al., 2019; Hajli, 2020; Leong et al., 2020). Consumer trust in social commerce is determined by their positive subjective beliefs that sellers and SC community members are sincere and offer reliable user-generated content. Hence, trust in sellers and SC community members are the two constructs that are employed to represent the people dimension of social commerce trust.

#### The Technology Dimensions

The idea of technology has typically been characterised as a component of trust (McKnight et al., 2002; Kim et al., 2005; Li et al., 2008; Lin et al., 2019). According to existing findings, the technical capabilities of social commerce advance from the SC platforms, including social media and e-commerce (Lin et al., 2019; Hajli, 2020; Tuncer, 2021), online payment platforms (Williams, 2021), and Internet-related SA (McKnight et al., 2002; Sarkar et al., 2020). These social commerce technological tools collaborate to offer assistance on social commerce attributes, online purchases, and social commerce behaviour of customers such as ratings, reviews, and sharing. According to this viewpoint, in the environment of social commerce tools, and trust, especially in social commerce, is the primary element that induces trust in the attitudes of customers.

First, based on the SC studies that have evaluated trust, social media offers fundamentals for the technological competence of social commerce (Ng, 2013; Lin et al., 2019; Hajli, 2020; Tuncer, 2021). Furthermore, trusting e-commerce websites add to the technical dimension of social commerce (Lin et al., 2019). In this research, trust refers to the opinions of people about certain e-commerce social networking sites. The primary element of trust in social commerce is trusting the SC platforms for e-commerce and social networking sites to gather the trust of customers in general SC, as employed by our research. Second, trusting online payment platforms facilitates the technological dimension of social commerce, especially in our social commerce research. In the conventional e-commerce environment, trust in online payment has been recognised as an important customer trust factor (Gong et al., 2020). It has also been included in customer trust in social commerce (Leong et al., 2021; Williams, 2021). Trust in online payment is a critical factor representing the trust beliefs of customers in social commerce since online payment is essential in conducting SC operations (Williams, 2021).

Finally, SA, or trust in the Internet, has been recognised as a critical characteristic of technology in various online situations (McKnight et al., 2002; Li et al., 2008; Alkhalifah and D’Ambra, 2013; Khan et al., 2021). It has been proposed that the SA of the platform of the context has a greater impact on trusting beliefs (McKnight et al., 2002). In a mobile commerce context, SA (promises, guarantees, rules, insurances, and terms and conditions of a contract) indicates the honesty of the seller and aids in the development of trust in the system (Sarkar et al., 2020). Hence, social commerce trust from a technology viewpoint is aided by the trust beliefs of customers in the SA of an SC platform. To summarise, we utilise three constructs to represent the technological dimension of SC trust, i.e., trust in the SC platform, SA, and online payment.

## Research Model and Hypothesis Development

**FIGURE 3:**
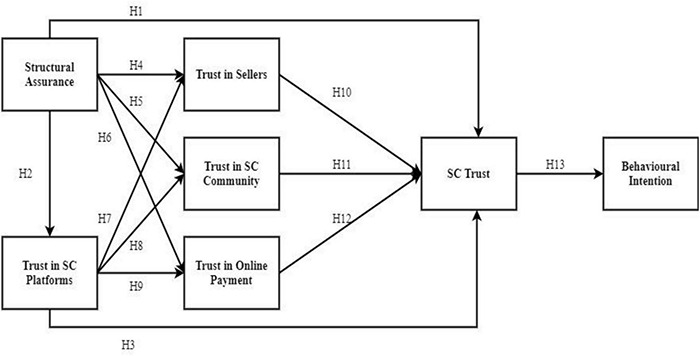
Research model.

Based on the social-technical theory and trust lenses, the proposed research model was developed as shown in Figure 3. The behavioural intention is determined mainly by trust in SC. Moreover, the trust in SC is affected by institution-based trust, trusting beliefs, and cognitive trust. The following sub- sections describe the research model and discuss the hypotheses development in detail.

### Institutional-Based Trust

The suggested model analyses the relation between trust in the SC platform and the Internet. According to cognitive dissonance theory (Bhattacherjee and Premkumar, 2004), individuals may be less inclined to provide their details if the environment that the Internet provides is considered unsafe, irrespective of the level of trust they have in a specific supplier. On SC platforms, the lack of face-to-face interaction generates confusion and insecurity (Featherman and Hajli, 2016). According to one study, trust in the Internet and e-commerce businesses is critical for making online purchases (Kim and Peterson, 2017).

Prior IT adoption research discovered a tangible link between SA and trust (Luo et al., 2010; Zhou, 2012; Oliveira et al., 2014; Khan et al., 2021). Moreover, mobile social commerce studies confirmed the substantial relationship between SA and SC trust (Sarkar et al., 2020). A significant relationship between social media interaction, SC applications and websites, and institution-based trust was discovered by Hajli (2020), leading to purchasing intentions on SC websites. Furthermore, SC trust is influenced by e-commerce and social networking sites (Lin et al., 2019). Likewise, SC users with a positive impression of SA and the SC platform are more inclined to share their data and utilise the SC. As a result, we predict that trust in the SC platform and SA influence the willingness of consumers to trust in SC. Accordingly, we posit the following hypotheses:

H1: Structural assurance has an influence on trust in social commerce.
H2: Structural assurance has an influence on trust in social commerce platforms.
H3: Trust in SC platforms has an influence on trust in social commerce.

Prior trust studies have shown that SA of the platform of the context has a more significant effect on trusting views (McKnight et al., 2002; Li et al., 2008; Khan et al., 2021). Moreover, SC consumers are more likely to have favourable, trusting attitudes in social commerce if they feel that sellers, SC communities, and online payment service providers on the Internet are run with truthfulness, sincerity, and compassion. In the context of technology, technological protections like encryption, particular system development methods, and techniques have been discovered to assist early trusting beliefs in an online seller and website (Li et al., 2008; Luo et al., 2010). Social commerce customers may also look for Internet rules and regulations and legal ways to secure their information (Jiang et al., 2021). We argue that trusting beliefs in online sellers, community members, and payment will enhance due to the SA connected to the Internet in general. As a result, we anticipate that users will be more likely to trust social commerce systems if they feel comfortable engaging with the Internet in general. As a result, the following hypotheses emerge:

H4: Structural assurance has an influence on trust in sellers.
H5: Structural assurance has an influence on trust in the social commerce community.
H6: Structural assurance has an influence on trust in online payment.

The safety, security, and guarantees provided by the e-commerce site or place of business of the seller form the basis of trust (Hajli, 2020). When customers trust an SC platform, they are more likely to purchase and engage in social interactions (Hajli, 2020; Tuncer, 2021). Individuals use SC platforms and make purchases when they feel the platforms are trustworthy and satisfy their needs (Rahman et al., 2020). Furthermore, the willingness of people to adopt social commerce platforms increases when their trust in the web vendors is positively influenced (Lu et al., 2016). Hence, we postulate that:

H7: Trust in SC platforms has an influence on trust in sellers.

Social commerce platform features such as reviews, forums, ratings, and communities are critical predictors of customer behaviour in the SC sector (Hajli and Sims, 2015; Cheng et al., 2021). Trust and perceived risks can be improved by online communities (Hajli, 2015). Trust can be maximised, and risks connected with online transactions can be minimised through interactions *via* the members and features of the online community on social commerce platforms and Internet technologies (Hajli, 2013; Rahman et al., 2020). Thus, we hypothesise:

H8: Trust in SC platforms has an influence on trust in social commerce community members.

Customers can make purchases employing online payment methods without revealing their personal information like credit card details by using several SC applications and websites available today (Mou and Benyoucef, 2021; Williams, 2021). Although Line, Facebook, Instagram, and other sites are primarily used for social networking, numerous commerce activities, like purchases, are also possible. Customer trust in the vendor as a prerequisite to purchasing decisions, evaluations, and shopping is becoming more critical as the market is brimming with purchase options (Hajli, 2020). Hence, customers are more likely to trust social commerce vendors and conduct online financial transactions if they have more vital institution-based trust in social commerce platforms.

H9: Trust in SC platforms has an influence on trust in online payment.

### Trusting Beliefs

Regarding the expertise, knowledge, and experience of social commerce sellers, buyers may be concerned about their competence and whether they are working for themselves or on behalf of someone else to meet their demands (Wongkitrungrueng and Assarut, 2020). By providing assistance in information and in notifying consumers that they care about their requirements, the generosity of sellers may be induced. Thus, they may promote their credibility by making good faith claims about the quality of the business offerings (items and services) they deliver to their consumers. Trust can assist SC consumers in overcoming their worries and encouraging them to use social commerce. Some businesses, such as Walmart, have failed to implement SC because of numerous security, privacy, and trust concerns (Kim and Park, 2013). Customers refrain from making e-commerce purchases by trusting the sincerity, competency, trustworthiness, and customer feedback of sellers (Hajli et al., 2017).

Trust is a challenging phenomenon in social commerce compared to traditional e-commerce (Gefen et al., 2003; Lin et al., 2019). Users interact with individual businesses under the social commerce C2C paradigm. The identities on both sides of the interaction are opaque, and there is a significant information imbalance (Leong et al., 2021). For example, sellers could make Facebook fan pages for managers to communicate directly with customers to enhance their relationships (Leong et al., 2021). Furthermore, seller and buyer interactions can be enabled through chat plug-ins in websites or chat applications, such as QQ, WeChat, etc. This can help customers receive more information and know the attitude, trustworthiness, and compassion of sellers to build their trust beliefs (Leong et al., 2020). Such factors make customers believe that purchasing decisions are risky where trust becomes visible and a severe issue (Tuncer, 2021). Hence, the ease and assurance required to make purchases through SC platforms rely on the level of trust of the seller (Wongkitrungrueng and Assarut, 2020). We propose the following hypothesis based on our analysis:

H10. Trust in sellers has an influence on trust in social commerce.

As a new e-commerce medium, social commerce incorporates social aspects and fosters consumer-generated material, such as suggestions, comments, images, reviews, and online streaming (Hajli and Sims, 2015; Cheng et al., 2021). Before making a purchasing decision, many customers seek the advice and suggestions of others (Chen et al., 2017). Social commerce offers significant benefits, such as linking merchants and consumers, and assisting them in acquiring and sharing product knowledge (Xiao et al., 2015).

In the context of interpersonal communication, social commerce networks may provide customers with a very engaging environment where they can trust and connect while also expressing their views on a product or service (Liang et al., 2011; Cheng et al., 2021). Trust in members is influenced by the social advantages of the community that customers acquire (Tsai and Hung, 2019). When consumers receive trustworthy, accurate, and timely information and tools from SC communities, their trust increases substantially (Yeon et al., 2019; Cheng et al., 2021). For a good shopping experience and better purchasing decisions, customers in SC depend on informative support and product reviews (Yang et al., 2021). According to Leong et al. (2021), online reviews significantly affect trust in mobile social commerce. Furthermore, Lin et al. (2019) discovered a strong relationship between social media elements (i.e., product ratings, reviews, and sharing) and social commerce trust. Hence, we hypothesise:

H11: Trust in the SC community members has an influence on trust in social commerce.

### Cognitive Trust

Online payment is critical to completing social commerce operations (Zhang and Benyoucef, 2016; Williams, 2021). Assumptions of customers that the SC online payment service features are credible are referred to as cognitive trust in the online payment (Leong et al., 2021). Previously, cognitive trust was defined as trusting attitudes involving trust in mobile and online payments (Gong et al., 2020; Leong et al., 2021). Trust in mobile social commerce is significantly influenced by cognitive trust in mobile payments (Leong et al., 2021). According to Lin et al. (2011), trust can be transmitted intra-channel when the trust of the customers is transferred to another entity within the shared channel. As both online payment and SC are part of the same online service-to-service intra-channel, we predict that the trust of customers in online payment will be extended to trust in social commerce services. Thus, the following is our hypothesis:

H12: Trust in online payment has an influence on social commerce trust.

### Trust in Social Commerce and Behavioural Intention

The extent to which an individual considers whether to indulge in a specific activity or the likelihood of the intention of an individual to perform a specific behaviour is known as behavioural intention (Venkatesh et al., 2013). This paper describes behavioural intention as the probability that people will use a social commerce site in the future to buy online. Customers who build trust in a particular SC platform are more likely to return (Meilatinova, 2021). According to Ng (2013), the intention of a customer to buy from a social commerce site is enhanced through trust in an SC platform. Moreover, a significant relationship exists between buying behaviour and social commerce trust. According to the results of a meta-analysis of SC research (Sarkar et al., 2020; Mou and Benyoucef, 2021), there is a substantial relationship between trust and customer behaviour intention.

As a multifaceted concept, social commerce trust impacts customer decisions as a holistic process that combines the cognitive and emotive processes by incorporating the effects of its aspects (McKnight et al., 2002; Lin et al., 2019). Customers with a high level of social commerce trust believe that SC platforms, online payment systems, and Internet technology are reliable and satisfy their needs during the online purchasing process. Consumers will also believe that vendors and SC communities are trustworthy and concerned about their requirements, leading to favourable impressions of trust in SC and a desire to continue using it. As a result, we propose the following hypotheses:

H13: Social commerce trust has an influence on behavioural intention.

### Control Variables

As control variables, this study includes demographic factors such as age, gender, and education. Prior empirical study has shown that individual user variations in behavioural intentions toward technology adoption are influenced by demographic factors (Venkatesh et al., 2012, 2013). Moreover, researchers have indicated that these variables are crucial to consider in customer surveys, emphasising the need to control them in uncertain behaviour, such as trust in social commerce (Booth and Nolen, 2012; Featherman and Hajli, 2016; Leong et al., 2020). Gender and age were found to influence trust in information technology by Pak et al. (2014). Furthermore, trust is significantly affected by education and gender (Calabrese et al., 2016). Based on prior findings, it seemed appropriate to include gender, age, and education as control variables in this study.

## Research Methodology

### Data Collection Procedure

This study uses a target sample of individuals who were older than 18 and had online shopping experiences through the SC platforms (e-commerce and SC websites or social networks platforms). Social networks sites (SNS), specifically, Facebook and Instagram users, were the target population. There are several reasons to choose this sample set. First, SNS is very popular, with millions of actual and potential SC users (Mou and Benyoucef, 2021). Second, Facebook and Instagram are among the top three SC providers with various online community and payment features and the highest SC users (Mou and Benyoucef, 2021). Third, different technical mechanisms and functionalities are conducted on both platforms. This is because they are attractive social networks, and it is crucial to investigate the key socio-technical differences. Furthermore, these socio-technical differences are significant to examine changes in beliefs and perceptions among responders (Dwyer et al., 2007), which enabled the demographic differences to construct the sample. Finally, research (Sibona and Walczak, 2012) reported that recruitment through SNS is suitable to gain insights into the behaviour of consumers and helpful in understanding the technology adoption. However, most previous SC adoption studies have focussed on Facebook as an SNS platform, and limited research has studied trust in social commerce from dual SNS platforms or different sites such as Instagram (Leong et al., 2020; Tuncer, 2021).

Facebook and Instagram are commonly used in almost every country in various languages. Moreover, data collection in different countries and cultures is challenging due to the language and cultural differences (Van de Vijver and Chasiotis, 2010). Therefore, this study is conducted in three English-speaking countries, namely, (i) Australia, (ii) the United States, and (iii) the United Kingdom. The selection of these countries is based on certain reasons. First, the survey was designed in English to ensure that the respondents can understand the questionnaire. Second, data collection from diverse settings could help to increase the generalisability of the research findings (Van de Vijver and Chasiotis, 2010). Third, Facebook and Instagram have been reported as the most popular social networks, with more users across the selected countries (Statista, 2021a,b).

Practically, collecting the information of Facebook and Instagram users is crucial. However, these platforms enable individuals to target users *via* advertising. Therefore, this study employs a banner advertisement placed through Facebook Adv and Instagram Adv as a technique to recruit subjects (Alvarez et al., 2003). This technique enables advertisers to determine which members view the ads by selecting target viewers based on age, gender, location, and interests. This study ran two banner campaigns through each site for 2 weeks, from 15 March 2021 to 30 March 2021.

In total, 843 people clicked on the advertisement and were directed to the survey website. The initial data analysis revealed that 258 (30.6% of the individuals directed to the survey website) did not complete the questionnaire and exited the survey at some point before completion, and only 585 individuals completed the questionnaire. Furthermore, knowing whether an individual has a prior online shopping experience with SC platforms, the filtering question of “Have you been involved in social commerce (e.g., buying online through social media) before?” was asked (Leong et al., 2020). Among 585 completed responses, some data were discarded. For example, 112 responses were ruled out since the participants did not have a prior online shopping experience with SC platforms. In addition, 67 individual responses were removed because they were invalid, either having the same answer to most questions or completing the survey in less than the average time (7 min). Therefore, 406 responses (usable data sets) were used for further analysis. The yielded sample size was adequate as it fulfilled the criteria for conducting confirmatory factor analysis using PLS (Goodhue et al., 2012; Hair et al., 2019).

### Items Scale

The measurement items were adopted from previous well-known researches to sustain construct and content validity (see Supplementary Appendix A). Gefen et al. (2011) argued that the total of three items loading on one construct should statistically identify the factor measurement. Accordingly, all constructs were measured by at least three items except for behavioural intention. The behavioural intention is defined as the extent to which individuals will continue purchasing using a particular SC platform in the future and is measured with five reflective items that have been modified and adapted from Bansal et al. (2010); Venkatesh et al. (2012), and Aren et al. (2013). Trust in SC was adapted from Kim and Park (2013). SA was adapted from McKnight et al. (2002). Trust in SC platform and trust in Sellers constructs (benevolence, competence, and integrity) were adapted from McKnight et al. (2002); Li et al. (2008), and Wongkitrungrueng and Assarut (2020). Trust in the SC community construct was adapted from Cheung et al. (2009); Hajli (2015), and Rahman et al. (2020), and trust in online payments items were adapted from Leong et al. (2021). A seven-point Likert scale that ranged from “strongly agree” to “strongly disagree” was used to measure the scales (Hinkin, 1998). A pre-test phase was considered to refine and validate the questionnaire (MacKenzie et al., 2011). The draft of questions and measurements was sent for review to 10 social networkers, who are online shoppers, and five information systems (IS) professors, seeking their confirmation of the face validity of the survey questionnaire. Based on their feedback, minor changes and revision were made.

### Common Method Bias

This study implemented some methods to assess and mitigate the influence of common method bias (CMB), as recommended by Barlette et al. (2021). *A priori* procedural remedy by Podsakoff et al. (2012) was included. This method was used during the pre-test phase to refine the scale items and to eliminate potential ambiguities, with multiple-choice questions periodically included to break up the pattern of questions rated using Likert scales. In addition, Harman’s single-factor test was conducted. The results of the Harman one-factor test showed that the total variance was less than 50%, indicating that CMB was not a concern in this study (Podsakoff et al., 2003). Furthermore, the path coefficients and correlation of the constructs obtained from the structural model assessment had varying degrees of significance (Barlette et al., 2021; Williams, 2021). These results suggested that CMB was not a problem in data.

In addition, a non-response bias test was conducted (Churchill, 1979). Two groups were formed according to the order in which participants completed the survey questionnaire. The first sub-sample consisted of the first 100 early respondents, while the second included the last 100 late respondents (Churchill, 1979). A two-tailed *t*-test at a 5% significance level was assessed to compare these two sub-sample groups (Field, 2009). The test findings showed no significant differences between these two groups of respondents. Hence, non-response bias was not a problem in this study.

## Data Analysis and Results

### Sample Statistics

Table 1 shows the demographic profile of respondents. The sample is majorly comprised of male respondents (55.6%). Moreover, in the sample, most of them were young, and the ages ranged from 18–41 (86.6%), with the remaining 13.4% older than 42 years. The subjects were relatively highly educated (85.5% had a Bachelor’s degree or higher). Concerning their online shopping history, 40% of the sample had frequently shopped online. The majority of respondents were mainly from the United States, Australia, and the United Kingdom, respectively.

**TABLE 1:** Demographic statistics.

Category	Frequency	Percent
**Gender**		
Male	226	55.6
Female	180	44.4
**Age (years)**		
Less than 18	0	0
18–21	60	14.8
22–31	138	34
32–41	154	37.9
42–51	31	7.6
52–61	16	3.9
More than 61	7	1.7
**Education**		
Not educated	0	0
High school	21	5.2
Diploma	35	8.6
Bachelor	245	60.3
Master	62	15.3
Ph.D.	40	9.9
Others	3	0.7
**Online Shopping Usage**		
Once a week	42	10.3
Twice a week	24	5.9
Several times a week	16	3.9
Once a month	67	16.5
Several times a month	166	40.9
Several times a year	79	19.5
Not specified	12	3
**Country**		
Australia	139	33.3
USA	147	36.2
UK	135	30.5

### Measurement Model

This study employs PLS using SmartPLS 3.0 software (Ringle et al., 2015) to analyse the data and to assess the structural relationships of the research model. This study employs the confirmatory composite analysis (CCA) approach, which is recently introduced and applied to confirm the measurement models when using PLS-SEM (Hair et al., 2020; Hair, 2021). This study follows the guidelines of Hair et al. (2020) in accessing PLS- CCA to confirm the reflective measurement model. Moreover, this study uses the PLS technique because it does not build a strict assumption about the normal data distribution and is preferable for composite analysis (Hair et al., 2021). In addition, non-parametric bootstrapping was carried out using 406 cases and 10,000 samples (Streukens and Leroi-Werelds, 2016) to obtain the significance of each structural path (i.e., the *t*-value) between the constructs (Hair et al., 2021).

This study is based on a structural model that contains six unidimensional (single) constructs (i.e., behavioural intention, SC trust, trust in SC community members, trust in online payment, and SA), two hierarchical (second-order) constructs (i.e., trust in SC platform and trust in sellers). Previous studies on trust in online contexts (McKnight et al., 2002; Li et al., 2008; Luo et al., 2010; Alkhalifah and D’Ambra, 2013) and social commerce (Lin et al., 2019) have specified these variables as a hierarchical construct consisting of few sub-dimensions and dimension in most cases.

#### First Order Construct

To verify the reliability of the measurement model, Composite reliability (CR), Rho-A, and Cronbach’s alpha (α) values were measured, as shown in Table 2. It has been suggested that these values should lie above 0.70 to demonstrate the reliability of the items (Henseler et al., 2016; Hair et al., 2020, 2021). The Composite reliability varied between 0.839 and 0.922, while Cronbach’s alpha values ranged between 0.770 and 0.905 for all variables. Thus, this confirms the reliability of the model. In addition, convergent validity and discriminant validity tests were carried out and were evaluated. For convergent validity, the factor loading value should be over 0.7, and the average variance extraction (AVE) should be around 0.5 (Henseler et al., 2016; Hair et al., 2020). Table 2 shows that AVE values were over 0.6 and factor loadings were above 0.770, exceeding the cut-off value. Therefore, these values validate the measurement model and indicate the possession of convergent validity. Table 3 also suggests that the square root of each AVE was greater than the relation between inter-structural values (Henseler et al., 2016; Hair et al., 2020).

**TABLE 2:** First-order construct descriptive.

Construct	Items	Loading	Cronbach’s Alpha	rho_A	Composite Reliability	Average Variance Extracted (AVE)
Behavioural intention (BI)			0.842	0.851	0.888	0.613
	BI1	0.770				
	BI2	0.831				
	BI3	0.829				
	BI4	0.771				
	BI5	0.708				
Cognitive trust -online payment (CTOP)			0.859	0.868	0.914	0.781
	CTOP1	0.916				
	CTOP2	0.909				
	CTOP3	0.823				
Structural assurance (SA)			0.840	0.843	0.904	0.759
	SA1	0.834				
	SA2	0.925				
	SA3	0.851				
Social commerce trust (SCT)			0.801	0.801	0.883	0.716
	SCT1	0.815				
	SCT2	0.888				
	SCT3	0.834				
Trust in SC community (TSCC)			0.802	0.805	0.883	0.716
	TSCC1	0.821				
	TSCC2	0.833				
	TSCC3	0.883				
Trust in SC platform -benevolence (TSCPB)			0.709	0.715	0.839	0.636
	TSCPB1	0.771				
	TSCPB2	0.878				
	TSCPB3	0.736				
Trust in SC platform- competence (TSCPC)			0.801	0.804	0.883	0.717
	TSCPC1	0.882				
	TSCPC2	0.859				
	TSCPC3	0.796				
Trust in SC platform- integrity (TSCPI)			0.829	0.831	0.898	0.746
	TSCPI1	0.824				
	TSCPI2	0.901				
	TSCPI3	0.865				
Trust in Sellers- Benevolence (TSELLB)			0.770	0.775	0.868	0.687
	TSELLB1	0.786				
	TSELLB2	0.896				
	TSELLB3	0.801				
Trust in sellers -competence (TSELLC)			0.811	0.816	0.889	0.727
	TSELLC1	0.879				
	TSELLC2	0.891				
	TSELLC3	0.785				
Trust in sellers- integrity (TSELLI)			0.829	0.835	0.898	0.746
	TSELLI1	0.815				
	TSELLI2	0.904				
	TSELLI3	0.870				

**TABLE 3:** Constructs correlation and square roots of AVE values.

	BI	CTOP	SA	SCT	TSCC	TSCPB	TSCPC	TSCPI	TSELLB	TSELLC	TSELLI
BI	**0.783**										
CTOP	0.686	**0.884**									
SA	0.738	0.679	**0.871**								
SCT	0.660	0.728	0.650	**0.846**							
TSCC	0.676	0.683	0.711	0.698	**0.846**						
TSCPB	0.708	0.669	0.665	0.672	0.678	**0.797**					
TSCPC	0.725	0.792	0.671	0.680	0.664	0.716	**0.846**				
TSCPI	0.653	0.599	0.666	0.657	0.687	0.619	0.626	**0.864**			
TSELLB	0.654	0.736	0.661	0.678	0.679	0.728	0.731	0.629	**0.829**		
TSELLC	0.691	0.764	0.700	0.708	0.703	0.672	0.836	0.669	0.732	**0.853**	
TSELLI	0.716	0.669	0.659	0.654	0.700	0.687	0.701	0.757	0.651	0.676	**0.864**

*BI, behavioral intention; CTOP, trust in online payment; SA, structural assurance; SCT, social commerce trust; TSCC, trust in SC community; TSCPB, trust in SC platform (benevolence); TSCPC, trust in SC platform (competence); TSCPI, trust in SC platform (Integrity); TSELLB, trust in sellers (benevolence); TSELLC, trust in sellers (competence); and TSELLI, trust sellers (Integrity).*

*The bold numbers are the square roots of the AVE values.*

In addition, a heterotrait-monotrait (HTMT) criterion test was assessed to test the discriminant validity (Hair et al., 2020). As presented in Table 4, all values are significantly less than 0.90, demonstrating that discriminant validity is established (Hair et al., 2020). Therefore, it can be argued that discriminant validity has been confirmed.

**TABLE 4:** Heterotrait-monotrait (HTMT) criterion.

	BI	CTOP	SA	SCT	TSCC	TSCPB	TSCPC	TSCPI	TSELLB	TSELLC
CTOP	0.797									
SA	0.873	0.796								
SCT	0.790	0.872	0.789							
TSCC	0.818	0.820	0.863	0.861						
TSCPB	0.901	0.858	0.867	0.890	0.899					
TSCPC	0.866	0.849	0.816	0.848	0.822	0.852				
TSCPI	0.780	0.708	0.798	0.804	0.841	0.809	0.769			
TSELLB	0.806	0.806	0.825	0.865	0.861	0.886	0.833	0.790		
TSELLC	0.818	0.811	0.847	0.878	0.866	0.887	0.891	0.817	0.827	
TSELLI	0.854	0.790	0.791	0.799	0.858	0.898	0.858	0.904	0.809	0.821

#### Higher-Order Constructs

This study estimated the measurement of higher-order constructs, that is, the second-order trust in the SC platform and trust in sellers. This study followed the repeated indicator approach to test the measurement proprieties of higher-order constructs [it has also been familiarised by a term called hierarchical component model (HCM)] (Lohmoller, 2013; Sarstedt et al., 2019). Its usage lies in the assessment of higher-order constructs. In PLS, HCM is deployed to compute a higher-order construct. For this sake, the score of lower-order construct is being utilised (Wetzels et al., 2009; Lohmoller, 2013; Sarstedt et al., 2019). Demonstrated variable indicators are usually exploited in this technique. Such variable quantities enable a researcher to acquire the results of lower-order constructs to incorporate them to achieve the high-end model design (Wetzels et al., 2009). Through the utilisation of this technique, the indicators are repeatedly availed. In the very beginning stage, the first-order latent development is done through the indicators to procreate the basic loadings, and then they are expanded to find out the second-order latent constructs (i.e., trust in SC platform and trust in sellers) for the sake of peripheral loadings (Wetzels et al., 2009; Sarstedt et al., 2019).

**FIGURE 4:**
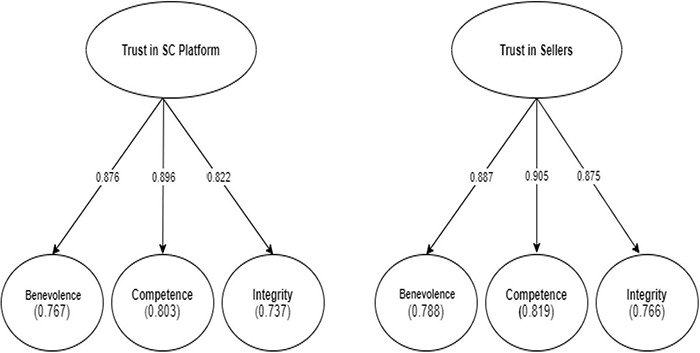
High constructs results.

This study assesses the correlations between the second-order variables and their first-order constructs (MacKenzie et al., 2011; Sarstedt et al., 2019). Table 5 and Figure 4 show that all path coefficients from the second-order factors to first-order constructs were significant at *p* < 0.001. The study confirmed that all loadings of the first-order latent factors on the second-order constructs (trust in SC platform and trust in Sellers) were above 0.70 (see Table 5 and Figure 3). Moreover, the study confirmed that Cronbach’s alpha, CRs, and AVEs were greater than 0.90 and 0.50, respectively, as shown in Table 3 (Henseler et al., 2016; Hair et al., 2020). Therefore, the reliability and validity of the higher-order constructs in the research model were supported.

**TABLE 5:** High-order construct descriptive.

Construct	Items	Loading	*T*-value	No of Items	Cronbach’s Alpha	rho_A	Composite Reliability	Average Variance Extracted (AVE)
Trust in SC platform (TSCP)				9	0.892	0.893	0.912	0.537
	TSCPB	0.876	67.470					
	TSCPC	0.896	94.552					
	TSCPI	0.822	63.663					
Trust in sellers (TSELL)				9	0.905	0.906	0.922	0.569
	TSELLB	0.887	74.981					
	TSELLC	0.905	100.261					
	TSELLI	0.875	71.028					

Furthermore, in Figure 4, the degree of explained variance of trust in the SC platform was reflected in terms of benevolence (77%), competence (80%), and integrity (74%). Similarly, trust in sellers was reflected in benevolence (79%), competence (82%), and integrity (77%). These findings supported our conceptualisation of the trust in the SC platform and trust in sellers as a higher-order structure.

### Hypothesis Testing

The results of the path analysis and hypothesis testing are presented in Table 6. The results show that among the 13 directly hypothesised relationships in the research model, two were non-significant.

**TABLE 6:** Hypotheses testing results.

Hypotheses	Association	Mean (M)	Standard Deviation (STDEV)	Path	T Statistics	*P* Values	Supported
				Coefficient			
H1	SA → SCT	0.029	0.057	0.031	0.546	0.585	No
H2	SA → TSCP	0.747	0.029	0.747***	26.043	0000	Yes
H3	TSCP → SCT	0.259	0.084	0.255**	3.019	0.003	Yes
H4	SA → TSELL	0.132	0.033	0.132***	4.056	0000	Yes
H5	SA → TSCC	0.296	0.061	0.297***	4.871	0000	Yes
H6	SA → CTOP	0.197	0.062	0.198**	3.217	0.001	Yes
H7	TSCP → TSELL	0.822	0.029	0.822***	28.332	0000	Yes
H8	TSCP → TSCC	0.526	0.058	0.525***	8.978	0000	Yes
H9	TSCP → CTOP	0.644	0.058	0.642***	11.136	0000	Yes
H10	TSELL → SCT	0.169	0.100	0.16	1.598	0.11	No
H11	TSCC → SCT	0.185	0.060	0.191**	3.152	0.002	Yes
H12	CTOP → SCT	0.25	0.059	0.256***	4.322	0000	Yes
H13	SCT → BI	0.63	0.036	0.628***	17.595	0000	Yes

**p < 0.01; ***p < 0.001.

Our findings suggest that the institution-based trust, SA (β = 0.031), has no influence on SC trust, whereas trust in SC platform (β = 0.255, *p* < 0.01) has a positive impact on SC trust. Therefore, H1 was not supported while H3 was supported. In addition, the proposed influence of SA on the trust in SC platform (H2) (β = 0.747, *p* < 0.001) was significant. SA was positively related to trust in sellers (β = 0.132, *p* < 0.001), trust in SC community members (β = 0.297, *p* < 0.001), and online payment (β = 0.198, *p* < 0.01) (H4, H5, and H6, respectively). Moreover, H7, H8, and H9 were all supported with the positive relationships found to be significant between trust in SC platform and trust in sellers (β = 0.822, *p* < 0.001), trust in community members (β = 0.525, *p* < 0.001), and online payment (β = 0.642, *p* < 0.001). The positive associations between trust in SC community (β = 0.191, *p* < 0.01) and online payment (β = 0.256, *p* < 0.001), and SC trust were significant, whereas the relationships between trust in sellers and SC trust (β = 0.16) were not significant. Thus H11 and H12 were supported, whereas H10 was not accepted. Finally, the hypothesised relationships related to the effect of SC trust on behavioural intention (H13) (β = 0.628, *p* < 0.001) were supported.

#### The Mediating Effects

The mediation effect of SC trust on the relationship between the dimensions of identified trust dimensions and behavioural intention was also tested. As this study does not approve of direct correlations between trust dimensions and behavioural intention, examining the mediating and indirect effects is appropriate (Tuncer, 2021). As shown in Table 7, trust in SC mediates the effect of SA (*t* value = 11.495, *p* < 0.05), Trust in SC platform (TSCP) (*t* value = 8.721, *p* < 0.001), Trust in SC community (TSCC) (*t* value = 3.308, *p* < 0.05), and Trust in online Payment (CTOP) (*t* value 4.288 = 0.03, *p* < 0.05) on behavioural intention. Nevertheless, TSC does not mediate the trust in the seller on behavioural intention. Therefore, these findings indicate that whereas trust has an indirect effect on behavioural intention through most of its dimensions, it has no indirect impact through other factors.

**TABLE 7:** The mediating effects.

Indirect correlation	Mediating	Standard Deviation (STDEV)	*T* values	*P* Values	Mediating effect
SA → BI	SCT	0.035	11.495	0.000	Yes
CTOP → BI	SCT	0.038	4.288	0.000	Yes
TSCC → BI	SCT	0.036	3.308	0.001	Yes
TSCP → BI	SCT	0.047	8.721	0.000	Yes
TSELL → BI	SCT	0.064	1.571	0.116	No

### Accessing the Structural Model

This study relied on Explained Variance *(R2)* to confirm whether the model achieved acceptable goodness of fit as illustrated; there was no other overall parametric criterion in PLS (Hair et al., 2019). The overall variance explained by the research model in terms of R2 was 0.463 for behavioural intention, 0.597 for SC trust, 0.638 for online payment, 0.637 for trust in SC community, 0.612 for trust in SC platform, and 0.849 for trust in sellers, as shown in Table 8.

**TABLE 8:** Explained variance and predictive relevance.

	R Square	Q^2^
BI	0.463	0.274
CTOP	0.638	0.490
SA	0.063	0.044
SCT	0.597	0.456
TSCC	0.637	0.447
TSCP	0.612	0.326
TSELL	0.849	0.479

This study also adopts Cohen’s (1988) effect size (*f2*) test for first-order constructs in the model. The sizes of effects are small, medium, and large with *f*2 values of 0.02, 0.15, and 0.35, respectively (Cohen, 1988; Sarstedt et al., 2017; Hair et al., 2019). The effect of size for SC trust on behavioural intention was high (0.702). Also, it was a large (1.34) adequate size for SA on trust in the SC platform. The effect size on the endogenous variable SC trust was highest for trust in the online payment (0.060), lowest for trust in the SC platform (0.26), and both indicated medium effects. The *f*2 effect size on the trust in seller was largest for trust in SC platform (1.73) and lowest for SA (0.046), indicating high and medium effects, respectively. Similarly, the effect size on the online payment was largest for trust in the SC platform (0.442) and lowest for SA (0.043), indicating high and medium effects, respectively. Moreover, the *f*2 effect size on the trust in the SC community was largest for trust in the SC platform (0.296) and lowest for SA (0.098), indicating medium and small effects, respectively.

This study evaluated further testation in this regard and rigorously determined the implications of predictive relevance (Q2). The predictive relevance, or the predictive sample reuse technique, is vital for testing the predictive validity of a complex model. It also helps measure how well-examined values are replicated by the model (Sarstedt et al., 2017). A blindfolding procedure was used with the omission distance of 7 to calculate Q2. This study obtains a cross-validated redundancy Q2 of all the focal variables, as shown in Table 8. The result of Q2 values exceeded zero (Q2 > 0), which revealed a highly predictive model (Sarstedt et al., 2017; Hair et al., 2021).

Furthermore, the PLS predicted algorithm was assessed to measure the predictive power (Shmueli et al., 2019). The predictive significance of the model was determined by comparing the mean absolute error (MAE), root mean square error (RMSE), and Q2 predict values for the PLS-SEM model against the naive benchmark model (LM) (Hair et al., 2019; Shmueli et al., 2019). Most indicators of the endogenous variables had positive Q2 values except BI1, COPT3, TSA1, TSCPC3, and TSELLC3. Moreover, the majority of indicators scored lower on the MAE in the PLS-SEM than the LM as shown in Supplementary Appendix B. This indicated that the structural model of this study shows a medium predictive power.

### Impact of Control Variables

This study has linked the control variables to all the endogenous variables in the model as recommended by Atinc et al. (2012) and Carlson and Wu (2012), with testing the impact of control variables. We assumed the path coefficient and *t*-value for endogenous variables, as shown in Table 9. The results yielded that five of 18 relationships (3 covariates* 6 dependent variables) were significant.

**TABLE 9:** Impact of control variables.

Control variable		BI	TSC	TSELL	TSCC	CTOP	TSCP
Age	*P* value	0.671	0.949	0.171	0.726	0.466	0.001***
	*T* value	0.425	0.065	1.368	0.350	0.729	3.435
Gender	*P* value	0.021*	0.177	0.473	0.615	0.065	0.207
	*T* value	2.306	1.350	0.717	0.504	1.848	1.263
Education	*P* value	0.005**	0.848	0.111	0.015*	0.086	0.000***
	*T* value	2.810	0.191	1.594	2.422	1.714	3.810

*p < 0.05; **p < 0.01; ***p < 0.001.

## Discussion

Previous research argues that the online environment is always impregnated with a sense of risk for users to opt for online activities and transactions (Featherman and Hajli, 2016; Williams, 2021). Risk is an essential factor affecting SC usage and behaviour (Mou and Benyoucef, 2021). Therefore, this study examines the role and the antecedents of trust in SC to manage the risk concerns. In doing so, it is aimed to understand the relationship between SC trust and its antecedents and behavioural intention. Based on social-technical theory and trust lens, we develop and test a research model.

The results confirmed that trust in SC was a significant determining factor for social commerce. It explained 46% of the variance in behavioural intention. This finding aligns with SC studies that revealed the critical role and effect of trust in SC (Lin et al., 2019; Hajli, 2020; Mou and Benyoucef, 2021; Tuncer, 2021). The results also showed that institution-based trust, i.e., trust in the SC platform, trust in the SC community members, and trust in online payment, had a significant effect on SC trust. These latent factors together explained 64% of the variance in trust in SC.

To confirm the reliability of an employing institution on SC, this study revealed that institution-based trust, i.e., SA and trust in the SC platform, is a salient antecedent to SC trust. Remarkably, there is no significant relationship between SA and SC trust. This result contradicts the findings of previous studies (Zhou, 2012; Oliveira et al., 2014; Sarkar et al., 2020). The possible explanation for the insignificant effect on the SC trust is that users may have a relatively clear knowledge about the soundness of the Internet as a platform and may have formed more specific risk or trust beliefs through which different transactions can be made. Another possible explanation was that consumers do not need to adopt SC infrastructure themselves because it is a task for the online platform that provides the SC service (Kong et al., 2020). This study has also found that SA impacted the trusting beliefs (trust in sellers, trust in SC community, and online payment). Our findings agree with previous studies that confirmed the relationships between SA and trusting beliefs (McKnight et al., 2002; Li et al., 2008; Khan et al., 2021).

This study showed that the second-order trust in the SC platform was reflected by the benevolence (β = 0.876), competence (β = 0.896), and integrity (β = 0.822) of the SC platform which explained 61% of the variance in trust in SC platform. The significant loadings of the three perceptions indicate that all of them hold capacity for trust in the SC platform. The results found out that trust in the SC platform has a highly significant effect on SC trust. This finding is consistent with recent studies in which trust in the SC platforms, such as social media and e-commerce sites, had played a direct role in SC (Lin et al., 2019; Hajli, 2020; Mou and Benyoucef, 2021; Tuncer, 2021). In addition, the analysis showed that trust in the SC platform had a high impact on trust in the seller, trust in the SC community, and trust in online payment. This finding complies with previously identified studies that SC platforms can optimise trust in sellers (Hajli, 2020; Tuncer, 2021), online communities (Hajli, 2013; Hajli and Sims, 2015; Cheng et al., 2019), and payment transactions (Gong et al., 2020; Leong et al., 2021; Williams, 2021). This study suggests that trust in SC platforms stemming from these important facets can significantly increase the total trust of potential consumers, enhancing positive behavioural intentions to adopt SC.

The second-order trust in seller analysis indicated that the trust in seller factor had three significant facets: benevolence (β = 0.815), competence (β = 0.904), and integrity (β = 0.870). Surprisingly, trust in sellers was not found to have an effect on SC trust. This discovery leads to a conflict with previous studies that shaped the trust of the consumer in online sellers (Luo et al., 2010; Kim and Park, 2013; Wongkitrungrueng and Assarut, 2020; Tuncer, 2021). This finding possibly reflects the perceptions of uncertainty and the shopping experience of consumers. Social commerce tenets permit the buyers to get connected with the seller. With such convenience, the buyer can get to know the seller, his/her proposed items, the quality and quantity, and each and everything can be discussed so that a buyer feels more comfortable carrying out the shopping process online rather than keep on negotiating physically with random sellers. This is how the propensity of social commerce is burgeoning, and more people are getting convinced of the fruitfulness of SC platforms (Wongkitrungrueng and Assarut, 2020). For example, one of the seller tools, live streaming, enables interaction and social presence, enhances the shopping experience, increases the level of trust that consumers have toward the online seller, and reduces their uncertainty (Hajli, 2015; Wongkitrungrueng and Assarut, 2020).

Trust in SC community members was found to be significantly affecting SC trust. This finding supports previous results in the literature (Cheng et al., 2019; Lin et al., 2019; Leong et al., 2020). Moreover, cognitive trust (trust in online payment) significantly impacted the SC trust. This finding agrees with previous empirical studies in suggesting that online payment plays a vital role in electronic commerce and social commerce trust formation and adoption (Gong et al., 2020; Leong et al., 2021; Williams, 2021).

This study confirms that demographic factors are not necessary to establish trust in social commerce. Well-educated gender and age groups were validated to have no confounding effect on trust in SC. This finding has provided a validation of previous research (Leong et al., 2020). The emergence of diverse social nexus and their technological attributes pertains to a disregard for the control groups. These escalated digital networks have led to the provision of public enlightenment and familiarity with online social commerce programmes. Such vast experience in the field of SC has enabled people to build their reliance on these tenets even though how knowledgeable the particular gender is and what is the age group (Leong et al., 2020). However, education was positively related to behavioural intention, SA, and trust in the SC platform and community. This indicated that educated subjects reported more trust and risk perceptions linked with the adaptability of SC. The possible explanation for this significant correlation was that our respondents were highly educated as 85.5% had a Bachelor’s degree qualification or higher. Therefore, this study confirmed that the education level of the SC users could play a significant role in the trust risk perceptions with different roles inherited with adopting SC (Calabrese et al., 2016; Featherman and Hajli, 2016). Finally, age significantly impacted trust in the SC platform, while gender positively influences behavioural intention. The latter finding supports previous studies and confirmed the effect of gender as control variables on user behaviours, in particular, affecting SC adoption (Jiang et al., 2021).

## Conclusion

### Theoretical Contribution and Practical Implication

#### Theoretical Contribution

This study provides several theoretical contributions and implications. First, this study offers a new conceptualisation of SC trust and demonstrates its significance by investigating trust factors and the relationships between its related antecedents, which has been limited in existing literature (Lin et al., 2017, 2019; Mou and Benyoucef, 2021). Our findings confirm the critical role of trust on behavioural intention. This study also identifies that institution-based trust, that is, trust in the SC platform, trusting beliefs in SC community members and features, and cognitive trust in online payment, are key predictors of trust in SC, therefore, confirming the critical role of these trust factors. By exploring and understanding the influencing factors of trust in SC, this study contributes to theoretical development in SC and its business outcome.

Second, this study contributes to and adopts the social-technical theory in the SC context. It also shows that both technological and social facets play essential roles in building trust in SC. A study by Lin et al. (2019) was the first to offer the implications of social-technical theory in the context of SC. They identified and examined four trust dimensions: trust in electronic commerce sites, trust in social media, consumers, and social commerce features. Their research suggested that future studies should consider an inclusive diversity of dimensions included when developing trust in SC. Accordingly, our study contributes to the literature by identifying various trust dimensions and empirically measuring their effects on SC trust involving trust in the Internet (SA), trust in SC platform, trust in sellers, trust in SC community, and online payment.

Third, this study enhances our understanding of trust formation in SC. Specifically, the direct effects of different trust factors were explored and empirically tested. Different trust factors have been addressed in SC literature (Lin et al., 2017; Sarkar et al., 2020; Mou and Benyoucef, 2021). However, the majority of existing SC studies have investigated trust from the one-dimensional concept. Limited research has addressed the trust in SC from a multi-dimensional viewpoint (Lin et al., 2017, 2019; Mou and Benyoucef, 2021). This study examined different trust dimensions, including single (first-order) and high (second-order) constructs, and the results confirmed and validated their conceptualisation. This study is one of the first to examine multi-dimensional trust constructs, exploring the relationship between them and their influence on trust in SC, confirming that trust plays a vital role in SC adoption and usage intention in the future.

Finally, this study does not emphasise a specific SC platform distinct from prior research that focussed on specific SC websites and addressed the limited setting (e.g., Mamonov and Benbunan-Fich, 2017; Lin et al., 2019; Hajli, 2020; Tuncer, 2021). In addition, it implied three different settings as the data were collected from three different countries. This is important to confirm the generalisability of the findings of the study. Therefore, this study provided a new contribution to the SC context research.

#### Practical Implication

This study provides an essential practical insight on the significance of building institution-based trust, as there is limited SC research on an institution-based trust (Luo et al., 2010; Hajli, 2020; Mou and Benyoucef, 2021; Tuncer, 2021). Our empirical findings confirm the importance of SA and SC platforms on building trust toward sellers, community members, and their features, and online payments in an SC context. In addition, SA has a significant positive relation with trust in the SC platform. Therefore, SC platform designers and managers should develop common trust models with online policies. They should pay keen attention to the development of benevolence, integrity, and competence elements in SC websites and applications to build reliable and long-term trust relationships with consumers and SC providers where they can interact (McKnight et al., 2002). Competence could be developed by providing agreement statements, information sharing, and recommendations about products and services. In addition, SC providers could enhance their benevolence by providing the provision of information related to privacy and security risks. Moreover, SC platform designers could develop features and technologies by focussing on identity theft and fraud through intrusion prevention. For example, developing methods to strengthen encryption and designing secure authentication interfaces.

Moreover, our findings show that cognitive trust in online payment significantly influences trust in SC. Therefore, SC platform providers should ensure that SC users feel secure, incorporate payment features, and contribute to the rise of consistency of different payments methods and services to point out the technical faults during the payment transaction processes. Careful investigation and elimination of important related issues, such as offering service warranty and ensuring full refund for incomplete transactions could enhance the trust and reduce the risk of the SC users.

Finally, education level significantly influences behavioural intention and some trust constructs such as trust in the SC platform and community members. Therefore, this study suggests that it is not suitable for SC providers and operators to apply a “one size for all” strategy to enhance the levels of trust (Leong et al., 2021). Different well-documented agreements and strategies should be implied to address the individual differences and education levels that exist among people to increase the levels of trust in SC platforms. Therefore, designing more convenient features and ancillary tools should be considered to enhance the trust formation among non-educated, educated, and highly educated consumers. For instance, strengthening the designs of the SC platform about navigation and content, information quality and reputation, experience sharing facility, and increased source credibility could lead the SC operators to successfully evaluate the level of trust in educated customers.

### Limitation and Future Studies

This study has a number of limitations that could be further addressed in future research. First, trust in the SC platform was conceptualised and was tested through the limited dimensions of SC platforms (e-commerce and social media sites). This study had a shortcoming of differentiating trust issues in various SC platforms. Different SC platforms, such as e-commerce websites, social media platforms, and SC mobile applications, could be tested separately to investigate their effects on SC trust and behavioural intention. Second, trust in sellers was not found to be significant on trust in SC. This possibly reflected the limited assessment of this factor which was only evaluated concerning the integrity and reliability of the sellers. Incorporating and examining more factors toward trust in sellers, such as information technology affordance (Tuncer, 2021) and symbolic value (Wongkitrungrueng and Assarut, 2020), could enhance trust in sellers leading to the reliability of SC platforms. This is how purchasing can be made dynamic. Third, the selected subjects were limited to only three English-speaking countries. Therefore, future research could evaluate the model in different settings and countries to confirm the generalisability of the findings as cultural differences exist among countries, which lead to different perceptions and behaviours (Venkatesh et al., 2012). Fourth, only trust in SC was measures concerning behaviour intention, which yielded 46% of explained variance. Future studies may test the moderation effects and incorporate other variables such as social factors, characteristics of individuals (e.g., interpersonal trust), privacy concerns, and design features into the model. After testing their direct, mediation, and moderation effects on both behavioural intention and trust in SC, new doors to the success of the SC industry can be brought.

Finally, this study evaluated and confirmed the best fit of the research model using a single SEM-PLS analysis method. Future studies may confirm the model validity and predict the performance of the variables using a dual-approach analysis employing machine learning tools such as artificial neural networks (ANN). Dual-approach (PLS_ANN) analysis has been recently suggested in social commerce (Leong et al., 2020) and information technology adoption research (Alwabel and Zeng, 2021).

### Conclusion Remark

Due to the increased number of social commerce users, existing researches have been conducted to consider the consumer behaviour on different SC websites. However, research paid little attention to the investigation of multi-facets trust attributes in social commerce. Hence, this study developed a research model based on social-technical theory and identified different trust facets to examine the importance of trust dimensions in social commerce. Using online surveys and data (*N* = 406) from three different countries, the empirical results contribute to theory and practice by explaining the relationships identified in the model. The findings of this study enhance our understanding of SC trust and open new debate in social commerce research.

## Data Availability Statement

The raw data supporting the conclusions of this article will be made available by the authors, without undue reservation.

## Ethics Statement

The studies involving human participants were reviewed and approved by the Committee of Health Research Ethics, Deanship of Scientific Research, Qassim University. The patients/participants provided their written informed consent to participate in this study.

## Author Contributions

AA was the principal investigator of the study, conceptualization to the data analysis, and conducted the experiments.

## Conflict of Interest

The author declares that the research was conducted in the absence of any commercial or financial relationships that could be construed as a potential conflict of interest.

## Publisher’s Note

All claims expressed in this article are solely those of the authors and do not necessarily represent those of their affiliated organizations, or those of the publisher, the editors and the reviewers. Any product that may be evaluated in this article, or claim that may be made by its manufacturer, is not guaranteed or endorsed by the publisher.
