# Supplementary material for: Exploring Trust Formation and Antecedents in Social Commerce
Source: Front Psychol. 2022 Jan 28;12:789863. doi: 10.3389/fpsyg.2021.789863 (PMC8831378; doi:10.3389/fpsyg.2021.789863)
Supplement: Supplementary file 1 [file Table_1.pdf]

Appendix: The study measurements.

Construct	Items	References
**Structural Assurance**	The Internet has safeguards to make me feel comfortable using it for SC.<br>I do not doubt the honesty of the Internet.<br>In general, the Internet is a robust and safe environment in which to complete online shopping transactions.	McKnight et al. 2002
**Trust In SC Platform**	**Benevolence**<br>I believe that a SC Platform would act in my best interest.<br>If I required help in purchasing, I would use a SC Platform.<br>A SC Platform would be interested in my well-being.<br>**Competence**<br>A SC Platform would be crucial in managing my online identity.<br>A SC Platform would manage my online shopping activities efficiently.<br>Overall, a SC Platform would be a capable and proficient online service provider.<br>**Integrity**<br>A SC Platform would be a truthful tool.<br>I would characterise a SC Platform as honest.<br>A SC Platform would keep its commitments	McKnight et al. 2002;<br>Li et al. 2008
**Trust in Seller**	**Benevolence**<br>I believe that the seller in SC Platform would act in my best interest.<br>If I required help, SC Platform sellers would do their best to help me.<br>A SC Platform seller would be interested in my well-being, not just only its benefits.<br>**Competence**<br>A SC Platform seller would be competent and effectively manage my online shopping.<br>A SC Platform seller would perform its role of managing my purchases efficiently.<br>Overall, SC Platform sellers would be capable and proficient online shopping providers.<br>**Integrity**<br>A SC Platform seller would be truthful in its dealings with me.<br>I would characterise SC Platform sellers as honest.<br>The seller in SC Platform would keep its commitments.	McKnight et al. 2002;<br>Wongkitrungrueng & Assarut, 2020
**Cognitive trust (online payment)**	Online Payment always provides accurate financial services.<br>Online Payment always provides reliable financial services.<br>Online Payment always provides safe financial services.	Leong et al., 2021
**Trust in SC community members**	I feel the recommendations by members of social commerce platforms are reliable.<br>I feel members on forums and community are reliable.<br>I think reviews and ratings on SC Platforms are credible.	Cheung et al. 2009; Hajli 2015 Rahman et al.,2020

**Social Commerce Trust**	I believe that social commerce will keep its promises and commitments.<br>Social commerce is trustworthy.<br>I would rate social commerce as honest.	Kim & Park, 2013
**Behavioural Intention**	I will continue using a SC Platform in the future.<br>I will always try to use a SC Platform in my online shopping activities.<br>I plan to continue using a SC Platform frequently.<br>I will likely provide the online vendor with the information to serve my needs through the SC platform better.<br>I am happy to use my credit card to purchase from an online vendor through the SC platform.	Bansal et al.,2010<br>Aren et al., 2013;<br>Venkatesh et al.,2012
