# Supplementary material for: Exploring Trust Formation and Antecedents in Social Commerce
Source: Front Psychol. 2022 Jan 28;12:789863. doi: 10.3389/fpsyg.2021.789863 (PMC8831378; doi:10.3389/fpsyg.2021.789863)
Supplement: Supplementary file 2 [file Table_2.pdf]

Appendix B: PLSpredict assessment of the original model (PLS) vs. naive benchmark (LM)

	PLS		Q²_predict	LM		PLS-LM	
	RMSE	MAE		RMSE	MAE	RMSE	MAE
BI1	1.085	0.878	-0.003	1.084	0.88	0.001	-0.002
BI2	1.047	0.832	0.058	1.047	0.829	0	0.003
BI3	1.117	0.921	0.043	1.115	0.917	0.002	0.004
BI4	1.197	0.97	0.048	1.201	0.974	-0.004	-0.004
BI5	1.083	0.869	0.052	1.084	0.865	-0.001	0.004
CTOP1	1.323	1.159	0.034	1.323	1.158	0	0.001
CTOP2	1.235	1.014	0.033	1.235	1.011	0	0.003
CTOP3	1.125	0.866	-0.002	1.126	0.851	-0.001	0.015
TSA1	1.158	0.938	-0.011	1.152	0.939	0.006	-0.001
TSA2	1.208	0.901	0.043	1.208	0.889	0	0.012
TSA3	1.073	0.867	0.069	1.063	0.861	0.01	0.006
TSC1	1.202	0.992	0.024	1.204	0.982	-0.002	0.01
TSC2	1.178	0.966	0.009	1.18	0.967	-0.002	-0.001
TSC3	1.146	0.856	0.018	1.148	0.855	-0.002	0.001
TSCC1	1.152	0.905	0.018	1.149	0.902	0.003	0.003
TSCC2	1.089	0.886	0.099	1.082	0.864	0.007	0.022
TSCC3	1.307	1.065	0.038	1.307	1.068	0	-0.003
TSCPB1	1.06	0.863	0.024	1.061	0.866	-0.001	-0.003
TSCPB2	1.077	0.892	0.017	1.074	0.887	0.003	0.005
TSCPB3	1.141	0.921	0.063	1.14	0.911	0.001	0.01
TSCPC1	1.178	1.015	0.023	1.181	1.018	-0.003	-0.003
TSCPC2	1.224	1.019	0.04	1.228	1.015	-0.004	0.004
TSCPC3	1.115	0.876	-0.01	1.113	0.88	0.002	-0.004
TSCPI1	1.045	0.828	0.099	1.037	0.804	0.008	0.024
TSCPI2	1.187	0.972	0.035	1.19	0.977	-0.003	-0.005
TSCPI3	1.057	0.85	0.06	1.057	0.847	0	0.003
TSELLB1	1.216	0.992	0.011	1.212	0.996	0.004	-0.004
TSELLB2	1.158	0.964	0.023	1.162	0.967	-0.004	-0.003
TSELLB3	1.189	0.985	0.032	1.193	0.98	-0.004	0.005
TSELLC1	1.304	1.148	0.021	1.303	1.15	0.001	-0.002
TSELLC2	1.081	0.911	0.041	1.081	0.908	0	0.003
TSELLC3	1.177	0.962	-0.045	1.155	0.936	0.022	0.026
TSELLI1	1.222	1.015	0.056	1.221	1.005	0.001	0.01
TSELLI2	1.138	0.912	0.055	1.135	0.9	0.003	0.012
TSELLI3	1.088	0.891	0.046	1.088	0.887	0	0.004
